# Natural Formulations: Novel Viewpoint for Scleroderma Adjunct Treatment

**DOI:** 10.1155/2021/9920416

**Published:** 2021-06-24

**Authors:** Shirin Assar, Hosna Khazaei, Maryam Naseri, Fardous El-Senduny, Saeideh Momtaz, Mohammad Hosein Farzaei, Javier Echeverría

**Affiliations:** ^1^Clinical Research Development Center, Imam Reza Hospital, Kermanshah University of Medical Sciences, Kermanshah, Iran; ^2^Pharmaceutical Sciences Research Center, Health Institute, Kermanshah University of Medical Sciences, Kermanshah 6734667149, Iran; ^3^Biochemistry Division, Chemistry Department, Faculty of Science, Mansoura University, Mansoura 35516, Egypt; ^4^Medicinal Plants Research Center, Institute of Medicinal Plants, ACECR, Karaj, Iran; ^5^Toxicology and Diseases Group (TDG), Pharmaceutical Sciences Research Center (PSRC), The Institute of Pharmaceutical Sciences (TIPS), and Department of Toxicology and Pharmacology, School of Pharmacy, Tehran University of Medical Sciences, Tehran 1417614411, Iran; ^6^Gastrointestinal Pharmacology Interest Group (GPIG), Universal Scientific Education and Research Network (USERN), Tehran, Iran; ^7^Departamento de Ciencias del Ambiente, Facultad de Química y Biología, Universidad de Santiago de Chile, Santiago, Chile

## Abstract

**Background:**

Scleroderma is a complex disease involving autoimmune, vascular, and connective tissues, with unknown etiology that can progress through any organ systems.

**Objective:**

Yet, no cure is available; the thorough treatment of scleroderma and current treatments are based on controlling inflammation. Nowadays, medicinal plants/natural-based formulations are emerging as important regulators of many diseases, including autoimmune diseases. Here, we provided an overview of scleroderma, also focused on recent studies on medicinal plants/natural-based formulations that are beneficial in scleroderma treatment/prevention.

**Methods:**

This study is the result of a search in PubMed, Scopus, and Cochrane Library with “scleroderma”, “systemic sclerosis”, “plant”, “herb”, and “phytochemical” keywords. Finally, 22 articles were selected from a total of 1513 results entered in this study.

**Results:**

Natural products can modulate the inflammatory and/or oxidative mediators, regulate the production or function of the immune cells, and control the collagen synthesis, thereby attenuating the experimental and clinical manifestation of the disease.

**Conclusion:**

Natural compounds can be considered an adjunct treatment for scleroderma to improve the quality of life of patients suffering from this disease.

## 1. Introduction

Within the human body, the immune system is considered one of the most complex biological systems [1], playing a fundamental role in protecting the body against detrimental effects of microbial pathogens and malignant tumor by various mechanisms such as engulfing, modulating, and moderating [2–4]. As a result, infectious diseases are prevented, and tissue or organ damages are abrogated. Aberrant immune responses may lead to autoimmune diseases [5] and destroy body cells [6]. Up to date, a broad range of chemical medications with well-known side effects are prescribed to manage the immune responses in immune pathology associated with infections, graft versus host disease, and hypersensitivity immune reactions (immediate or delayed-type) and especially for the treatment of autoimmune diseases [7]. Besides that, plant species with a broad spectrum of phytochemicals, fewer side effects, better compatibility, greater accuracy, low cost, and easy availability are frequently used to manage a wide range of diseases [8, 9]. Herbal formulations and their bioactive metabolites were found effective to mediate the proper functionality of the immune system through both immunosuppressive and immunomodulatory activities [2, 10–12], making them remarkable candidates for the treatment of immune-mediated disorders including autoimmune diseases and organ transplant rejection [13]. It was reported that several pure bioactive compounds of *Ganoderma lucidum* (Curt.: Fr.) P. Karst. (Aphyllophoromycetideae), *Panax ginseng* C.A. Mey. (Araliaceae), and *Zingiber officinale* Roscoe (Zingiberaceae) possessed immune cell-stimulating activity [3]. The immunosuppressive properties of isogarcinol, a natural compound from *Garcinia mangostana* L. (Clusiaceae), could serve as a new oral immunomodulatory drug for preventing transplant rejection and for long-term medication in autoimmune diseases [14]. It was proposed that herbal immune-stimulator compounds were able to modulate the innate immune response in fish and shellfish diseases [9]. The significant effect of *Salvia miltiorrhiza* Bunge (Lamiaceae) on the reduction of the inflammatory cytokines and mediators was proven against acute graft rejection and autoimmunity diseases [13]. This review tries to highlight the efficacy of medicinal plants on scleroderma treatment. Besides, we provided a brief introduction to autoimmune diseases and beneficial herbal plants for the treatment of such disorders.

### 1.1. Autoimmune Diseases

Although autoimmune diseases are rare, approximately 7.6–9.4% of the world population is affected by autoimmune inflammatory diseases. However, the incidence and prevalence of various autoimmune diseases are rising in women [6]. In addition to being a cause of mortality all around the world, autoimmune diseases are accompanied by severe chronic morbidity in patients' life including pain, inflammation, and tissue damage [15, 16]. Intense lifestyle and constant medical services for patients with autoimmune diseases are a huge burden for public health and the economy [17]. The description of autoimmune diseases requires a brief explanation of the immune system.

### 1.2. Current Treatment for Autoimmune Disease

Traditional medications for autoimmune inflammatory diseases include nonsteroidal anti-inflammatory drugs (NSAIDs), glucocorticoids, and disease-modifying antirheumatic drugs (DMARDs). NSAIDs are often prescribed for the treatment of arthritis and headaches because they possess analgesic, antipyretic, and anti-inflammatory effects [18] and can reduce pain through blocking cyclooxygenase (COX) enzymes [19]. NSAIDs are including traditional nonselective NSAIDs and selective COX-2 inhibitors [20]. Despite the diversity in their chemical structures, NSAIDs can inhibit autoimmune inflammatory responses [18]; however, they suffer from adverse effects (i.e., on the cardiovascular system) [21, 22]. Glucocorticoids such as prednisone/prednisolone, methylprednisolone, and fluorinated glucocorticoids such as dexamethasone and betamethasone are more frequently used for the treatment of severe rheumatoid arthritis since 1940 [23–25]. Glucocorticoids suppress cellular signaling pathways such as activator protein 1 (AP-1) and nuclear factor kappa-light-chain-enhancer of activated B cells (NF-*κ*B), as they can bind to specific receptors, and ultimately regulate the expression of cytokines and chemokines [26]. Furthermore, it has been reported that glucocorticoids can inhibit the proliferation of effector T cells [27]. Despite the high efficiency of glucocorticoids in the treatment of chronic diseases, their undesirable effects including gastrointestinal ulcers and bleeding, infection, immunosuppression, and bone damage are undeniable [28]. To alleviate inflammation in autoimmune diseases, several medications with diverse chemical structures were developed as DMARDs, which include methotrexate, leflunomide, gold compounds, sulfasalazine, azathioprine, cyclophosphamide, antimalarials, D-penicillamine, and cyclosporine [29]. Antitumor necrosis factor (anti-TNF) biologics or combination therapy of conventional disease-modifying antirheumatic drugs (cDMARDs) [16, 30], corticosteroids, anticytokine therapies, physical therapy, inhibition of intracellular signaling pathways, costimulation inhibition, biological inhibitors of T cells, B cell energy and depletion, regulatory T cells, and stem cell transplantation are other current treatments for patients with autoimmune diseases [31]. Considering their adverse effects, the introduction of novel strategies with fewer or no life-threatening adverse effects and lower toxicity with high efficacy seems essential [5]. To this notion, two different approaches were assumed: behavioral modification, which is an impact factor to suppress the onset of some autoimmune diseases or reduce their frequency, and the discovery of new drugs to inhibit the autoimmune diseases at early stages, rather than just controlling the symptoms [15]. Thus, usage of plant origin active substances in human diet might be an effective approach to regulate immune diseases and to maintain the body health [4].

### 1.3. Scleroderma

Scleroderma is an autoimmune disease. The name comes from SCLERO (hardness) and DERMA (skin). Scleroderma is characterized by typical changes in the skin (becomes hard locally or all over the body) and may affect visceral organs including kidneys, lung, heart, gastrointestinal tract, and skeletal muscles. It involves the accumulation of collagen leading to skin fibrosis. It can be classified into local or systemic (affects not only the skin but also other organs such as the lung) [32]. In the North American population, around 443 cases/10^6^ are diagnosed with scleroderma [33]. The prevalence, incidence, and clinical features of the disease change per the geographic place, where the severity and complication of scleroderma were higher in African-Americans in comparison with Caucasians due to the difference in the autoantibodies that were detected [34–39]. An Asian-Indian study demonstrated that the disease is more prevalent in younger ages [39]. Women are more susceptible to scleroderma than men (in the range of 3 : 1 to 14 : 1), indicating probable involvement of sex hormones (i.e., estrogen) in the development of scleroderma [40]. Estrogen has been found to regulate the extracellular matrix (ECM) components and the cell adhesion molecules in fibrosis [41, 42]. Considering the speed of progression, severity of skin hardening, and involvement of visceral organs, there are two main subdivisions of systemic sclerosis: limited cutaneous (lc-SSc) and diffuse cutaneous (dc-SSc). In lc-SSc, the fibrosis is limited to the arms, hands, and face, whereas in dc-SSc, the fibrosis could reach the heart, lung, and kidney [43, 44].

### 1.4. Pathogenesis of Scleroderma

**Figure 1:**
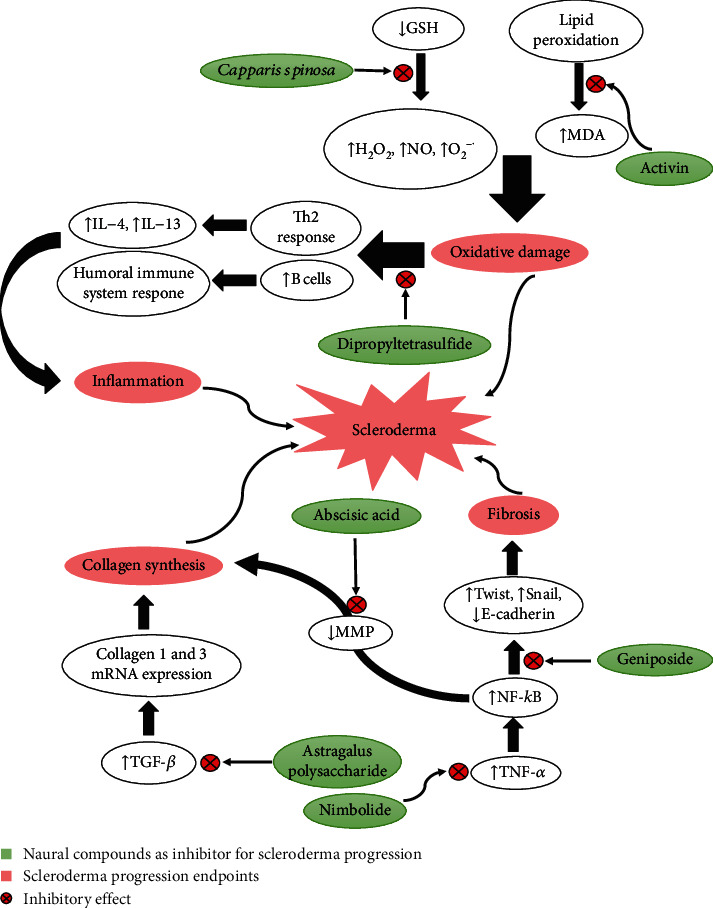
Pathogenesis of scleroderma; some natural compounds can inhibit its progression.

Different factors have been reported to play roles in scleroderma onset or progression, in which alterations in the immune components, exposure to toxins, genetic mutations, and oxidative stress are the most important participants (Figure 1).

### 1.5. Immune System and Scleroderma

B cells produce autoantibodies against different cellular organs such as anti-centromere, anti-topoisomerase-1, and anti-RNA polymerase III, which may progress skin fibrosis and tighten. The interplay between major histocompatibility complex (MHC) in dendritic cells and T cell receptors leads to the expression of anti-inflammatory cytokines (interleukins 4 and 13 (IL-4 and IL-13)) and the activation of B cells to secrete vascular epidermal growth factor (VEGF) [45]. Patients with scleroderma were shown to have higher levels of rheumatoid factors (RF), cryoglobulins, and *γ*-globulin autoantibodies [46]. Upregulation of coreceptor cluster of differentiation-19 (CD19), CD21, CD86, and CD95 on memory B cells elevates the autoantibody production against endothelial and fibroblast cells. The binding of endothelial and fibroblast autoantibodies with their antigen induces the production of reactive oxygen species (ROS) and apoptosis [47, 48]. The most dominant type of T cells is CD4^+^ type over CD8^+^ one [49]. The T cell receptor type is *γ*:*δ* showing *Vδ*1 chains [50]. The skin is composed of three layers: the outermost layer of skin is called the epidermis, the layer beneath it is called the dermis, and the deeper layer is called subcutaneous or hypodermis. The infiltration of T cell subsets is different between the layers of the skin. The avian model of systemic sclerosis showed infiltration of the *γ*:*δ* T cell, while the dermis and subcutaneous were infiltrated with the other subtypes of T cells (*α*:*β* T cell) [51]. Infiltration of CD14^+^ monocytes/macrophages was detected in early diagnosed patients with systemic sclerosis [52]. In addition to that, the degranulated mast cells increased in scleroderma patients at early stages [53]. The imbalance in the level of cytokines has been implicated with the pathogenesis of scleroderma. Higher levels of the inflammatory cytokines such as interferon gamma (INF-*γ*), colony-stimulating factors (CSF), IL-1, IL-4, IL-17A, IL-6, IL-13, IL-12 [54], IL-23 [55], IL-27 [56], transforming growth factor beta 1 (TGF-*β*1), T helper 2 (Th2) cytokine, and the chemotaxis monocyte chemoattractant protein 1 (MCP-1) were detected in scleroderma patients. An increase of Th2 cytokines leads to collagen synthesis, differentiation of fibroblasts to myofibroblast phenotype, and production of TGF-*β*, inducing the ECM remodeling [57]. Additionally, the infiltration of CD8^+^ and CD4^+^ T cells, activated macrophages, and human leukocyte antigen-DR isotype (HLA-DR) has been manifested in scleroderma patients [58]. Usually, this infiltration is observed in the early stages of the disease, which are diminished later. However, some patients with a longstanding history of the disease showed infiltration of immune cells in skin lesions. Most of these cytokines are associated with fibrosis of the skin and lung as well as with a higher level of autoantibody production against topoisomerase I [58].

### 1.6. Signaling Pathways in Scleroderma

#### 1.6.1. Platelet-Derived Growth Factor Pathway

The predominant manifestation of scleroderma is associated with dysregulation of the immune system, cytokine production, and production of collagen and its deposition. The growth and proliferation of connective tissue cells are regulated by the level of growth factors such as platelet-derived growth factor (PDGF) and its receptor (PDGFR). The binding of PDGF with its receptor induces the expression of extracellular matrix components such as collagen [59, 60]. Immunostaining of skin biopsy from scleroderma patients showed that there is a high level of PDGF-beta receptor in their skin [59]. This was correlated with a previous finding for the higher serum level of *β*-thromboglobulin. It has been reported that *β*-thromboglobulin is colocalized with PDGF in the granules of platelets, which may contribute to the higher level of PDGF in scleroderma patients [60–62]. An early pathological alteration in the scleroderma is vascular damage, where the function of endothelial cells and the ultrastructure of microvessels are altered [48, 63].

#### 1.6.2. Transforming Growth Factor Pathway

TGF-*β* is a key regulator for the signaling pathway that secrete the components of the extracellular matrix. The correlation between the severity of the scleroderma and the level of TGF-*β* is controversial. A low level of TGF-*β*1 was detected at the early stages of scleroderma; however, higher levels of TGF-*β*1 are responsible for the disease prognosis [64, 65]. Moreover, Wakhlu et al. correlated interstitial lung fibrosis with an elevated level of TGF-*β*1 and other cytokines [58]. Upon binding of TGF-*β*1 to its receptor, the intracellular cytoplasmic Smad3 is phosphorylated and translocated to the nucleus, leading to the transcriptional activation of genes involved in ECM remodeling [66]. It was reported that fibroblasts derived from systemic sclerosis contain a high level of pSmad3 and more DNA-binding affinity [67, 68]. Targeting the TGF-*β*1 signaling may provide a reliable therapeutic approach for the treatment of skin fibrosis in scleroderma patients. It was shown that inhibition of TGF-*β*1 by Repsox attenuated skin fibrosis *in vitro* and *in vivo* (bleomycin-treated mice) through downregulation of the connective tissue growth factor [68].

#### 1.6.3. NOTCH Pathway

The NOTCH proteins are required for cell proliferation, fate, differentiation, and death. NOTCH is a unique pathway, since both the ligand and the receptor are transmembrane molecules (called juxtacrine). The ligands for the NOTCH receptor (signal-receiving cell) are expressed on neighboring cells (signal-emitting cell) and known as Delta (called Delta-like in humans) and Serrate (called jagged in humans) [69]. The binding of the ligand with the extracellular domain of NOTCH leads to the activation of endocytosis by signal-emitting cells causing the proteolytic cleavage of the extraocular domain by the *α*-secretase enzyme (called 1^st^ cleavage). After that, a second cleavage occurs in the intracellular domain by *γ*-secretase to release the NOTCH intracellular domain (NICD). NICD then is translocated to the nucleus to release transcriptional repressors and to activate the gene transcription [70, 71]. There is evidence that NOTCH proteins are involved in the regulation of fibrosis and physiology and function of the vascular system [72, 73]. The polymorphism in *NOTCH4* (rs443198 and rs9296015) gene has been associated with systemic sclerosis [74]. Moreover, a missense mutation in the *NOTCH4* gene (chromosome 6p21 locus, c.4245G > A: p.Met1415Ile) was identified by a family-based whole exosome sequencing study and was linked to the pathogenesis and development of systemic sclerosis in this family [75, 76]. Strong immunostaining of NICD and overexpression of the HES-1 gene were detected in dermal fibroblasts of systemic sclerosis patients. Additionally, infiltrated lesions in the same skin biopsies represented a selective staining pattern against the ligand jagged canonical notch ligand 1 (JAG1) on T cells. This may indicate the possible interaction between T cells and the dermal fibroblasts, which leads to the overexpression of type 1 collagen and differentiation of fibroblast into myofibroblast (higher level of alpha-smooth muscle actin (*α*-SMA)) [77, 78]. Targeting the NOTCH pathway could be a valuable therapy for systemic and local sclerosis.

#### 1.6.4. JAK/Signal Transducer Activator of Transcription

Janus kinases (JAKs) are known as nonreceptor tyrosine kinases. They play a key role in the response to cytokines (such as IL-6) and growth factors. Upon binding of IL-6 to its receptor, JAK becomes activated and phosphorylates the cytoplasmic domain of the IL-6R at tyrosine residues. Signal transducer activator of transcription (STAT) is recruited at the phosphorylated tyrosine residue and dimerized upon phosphorylation. The dimer of STAT is translocated to the nucleus for activation of gene transcription [79]. Although dermal fibroblasts do not express IL-6R, a high level of soluble IL-6R can interact with IL-6, allowing the complex to bind to the fibroblast surface via glycoprotein 130 (gp-130) protein. Such binding activates the downstream STAT3 proteins and allows fibroblasts to differentiate to myofibroblasts, thus increasing the expression of type 1 collagen [66]. It was stated that the expression of STAT4 (rs7574685) changes in pulmonary fibrosis [74, 76]. Moreover, the knockdown of STAT4 protected the bleomycin-injected mice from the development of systemic sclerosis via reducing the T cell infiltration and the cytokine levels of tumor necrosis factor-alpha (TNF-*α*), IL-6, IL-2, and INF-*γ* [80]. Targeting IL-6 in systemic sclerosis patients showed moderate clinical improvement, while selective targeting of its downstream kinases such as JAK could be a potential therapeutic approach [81, 82]. A recent finding by Wang et al. revealed that JAK1 and 3 and selective inhibitor tofacitinib [83] were able to inhibit skin and lung fibrosis in bleomycin-treated and noninflammatory fibrosis (tight skin 1 (TSK-1)) mouse model [84].

#### 1.6.5. Akt/PI3K/mTOR/HIF-1*α* Pathway

Akt or protein kinase B is involved in metabolism, proliferation, and cell survival. It is activated by insulin and growth factors via the activation of phosphoinositide-3-kinase (PI3K). The activation of Akt/PI3K leads to the activation of mammalian target of rapamycin (mTOR), which further increases the synthesis of proteins such as hypoxia-inducing factor-1*α* (HIF-1*α*) [85]. In normoxia, there is a low level of HIF-1*α* within cells, while it is rapidly degraded after translation. On the other hand, in hypoxic conditions, HIF-1*α* is activated. HIF-1*α* plays the main role in response to a hypoxic condition, resulting in ECM remodeling, and cytokine and growth factor secretions [86]. The skin of naïve scleroderma patients showed a high level of HIF-1*α* associated with the overexpression of VEGF [87].

#### 1.6.6. Mitogen-Activated Protein Kinase (MAPK) Pathway

The Ras/Raf/MEK/ERK pathway transfers the extracellular signal to the nucleus via a tyrosine kinase receptor. The output signal depends on the cell type [88]. Chen et al. revealed that in systemic sclerosis patients constitutive ERK is activated, which is characterized by the overexpression of profibrotic genes (CGGF) and syndecan 2 and 4 (heparan sulfate proteoglycans) [89]. The constitutive ERK activation was reported in lung fibroblast, and its inhibition by PD98059 reduced collagen production [90].

### 1.7. Extracellular Matrix and Scleroderma

The cells connect with each other from the same type (homophilic interaction) or with different cell types (heterophilic interaction). The interaction is controlled by different cell adhesion molecules (CAMs) such as the immunoglobulin-like superfamily, integrins, cadherin, and selectins. The trafficking of lymphocytes and immune cells in general is regulated by the extracellular matrix components [91]. In systemic sclerosis patients, the level of surface CAMs is reduced in comparison with an elevated level of soluble forms (circulating) such as the intercellular adhesion molecule 1 (ICAM-1), endothelial leukocyte adhesion molecule 1 (ELAM-1), vascular cell adhesion molecule 1 (VCAM-l), P-selectin, and E-selectin [92–94]. The higher level of CAMs and expression of procollagen led to fibrosis in systemic scleroderma patients (90.9%) [95], indicating the key role of CAMs in the induction of inflammation and infiltration of immune cells at the skin and internal organs.

### 1.8. Fibroblast and Scleroderma

Within the body, fibroblasts are the main producer of collagen. Fibroblasts are activated upon engagement with endothelial cells and the infiltrated immune cells. It has been reported that activated fibroblasts produce type I and II collagens in the microvasculature and the perivascular [96]. *In vitro* studies exhibited that fibroblasts from systemic sclerosis patients express high levels of *α*-SMA and CAMs (*α*v*β*3 and *α*v*β*5 integrin) that leads to sustained activation of the TGF-*β* pathway [97], as well as proinflammatory and chemotaxis cytokines such as IL-6, TNF-*α*, IL-1*α*, IL-1*β*, and MCP-1 [98–100]. Additionally, these fibroblasts resist Fas-mediated apoptosis [101]. Later, Samuel et al. showed that this apoptosis resistance might be due to a deficiency in acid sphingomyelinase [102]. Based on this information, activated fibroblasts are potential targets for treatment or attenuation of the complications of scleroderma.

### 1.9. Oxidative Stress and Scleroderma

Oxidative stress is a term used when there is an imbalance between antioxidants and reactive oxygen and nitrogen. Reactive oxygen species (ROS) include superoxide anion (O_2_^·^), hydrogen peroxide (H_2_O_2_), and hydroxyl radicals (^·^OH). Reactive nitrogen species (RNOS) include nitric oxide (^·^NO) and peroxynitrite (ONOO^ˉ^). In addition to that, the hypochlorous acid (HOCl) is produced by neutrophil by the action of myeloperoxidase [103, 104]. When the level of ROS, RNOS, and HOCl is higher than the capacity of the antioxidant system in the cells, oxidative stress happens. Oxidative stress attacks different cellular targets including lipids, proteins, DNA, and other biomolecules. There are enzymatic and nonenzymatic antioxidants that scavenge the radicals and inhibit cellular damage. Enzymatic antioxidants include superoxide dismutase, glutathione peroxidase, GSSG (oxidized glutathione) reductase, glutathione-*S*-transferase, and catalase. Nonenzymatic antioxidants include *α*-tocopherol and carotenoids [105]. Antioxidants quench free radicals and prevent deleterious damage to cellular molecules.

The link between oxidative stress and the pathogenesis of scleroderma was explained by Murrell for the first time [106]. It has been reported that ROS can activate the secretion of proinflammatory and profibrotic cytokines such as PDGF and TGF-*β*. In turn, these cytokines induce fibroblast differentiation into myofibroblasts, enhance the expression of type I collagen, and cause vascular damage [107–109]. Increased ROS in the serum of systemic sclerosis patients with pulmonary arterial hypertension could induce collagen type I promoter in vascular smooth muscle cells [110]. Application of mesenchymal stem cells overexpressing thioredoxin-1 restored skin morphology, endothelial cell function, and tubular formation in a bleomycin-induced mouse model via reduction of oxidative damage and inhibition of TGF-*β* and hypoxia-inducing apoptosis [111]. The administration of N-acetyl-L-cysteine (NAC) *in vitro* showed promising results in alleviating the symptoms of systemic sclerosis and idiopathic pulmonary fibrosis by removal of superoxide anions and peroxynitrite [112–115]. ROS was found to maintain the phosphorylated form of PDGFR and ERK via the inhibition of protein tyrosine phosphatase 1B (PTP1B) phosphatase activity through oxidation of cystine residue at the active site [116]. Probable benefits of antioxidants should be considered in systemic sclerosis treatment.

### 1.10. Apoptosis and Scleroderma

As mentioned, apoptosis is highly regulated by different genes such as Fas. Pathogenesis of scleroderma is somehow correlated with dysregulation in the apoptosis process. High percentages of apoptotic endothelial cells were found in the dermis of systemic sclerosis patients and local cutaneous sclerosis biopsies. An excessive level of antiendothelial cell antibodies (AECA) was detected in these cells, a piece of evidence that indicated apoptosis may be involved in the development of scleroderma [117]. In another study, bleomycin induced scleroderma via the overexpression of Fas and Fas ligand and by subsequent activation of caspase 3 and apoptosis in a mouse model [118]. The CD8^+^ T cell apoptotic rate was higher than that of CD4^+^ T cells in the systemic sclerosis patients due to a low level of NF-*κ*B transcription factor and increase of CD4^+^:CD8^+^ T cells [119].

### 1.11. MicroRNA and Scleroderma

MicroRNAs (miRNAs) are noncoding RNAs 15-22 nucleotides in length. They bind to the 3′-untranslated region of mRNA (3′-UTR), leading to repression of protein translation [120]. Under certain conditions, miRNAs can activate gene expression [121]. It has been reported that miRNAs shuttle between cellular compartments and can be excreted to the extracellular matrix [122]. miRNAs play an important role in animal development, and their dysregulations have been reported in different diseases [123, 124]. Alteration in the miRNA level is correlated with the pathogenesis of scleroderma [125]. Honda et al. showed that dermal fibroblasts express a low level of miR-196a, which leads to the overexpression of type I collagen [126], and this downregulation was mediated via the TGF-*β* pathway [126]. Furthermore, downregulation of let-7a miRNA induced fibrosis by excessive production of type 1 collagen [127]. The serum level and dermal fibroblast level of miR-92a were found to be high in systemic sclerosis patients, leading to downregulation of metalloprotease-1 and collagen accumulation [128]. Suppression of miR-150 was correlated with the clinical manifestation of systemic sclerosis patients. A low level of miR-150 led to the overexpression of *β*3 integrin, Smad3 phosphorylation, and upregulation of type 1 collagen [129]. Recent work by Nakayama et al. revealed that a balance between miR-4458 and miR-18a is required for collagen synthesis downregulation. Both miR-4458 and miR-18a are downstream targets of IL-23 cytokine [130]. This may indicate the potential use of anti-IL-23 in scleroderma treatment.

## 2. Methods

### 2.1. Search Strategy

**Figure 2:**
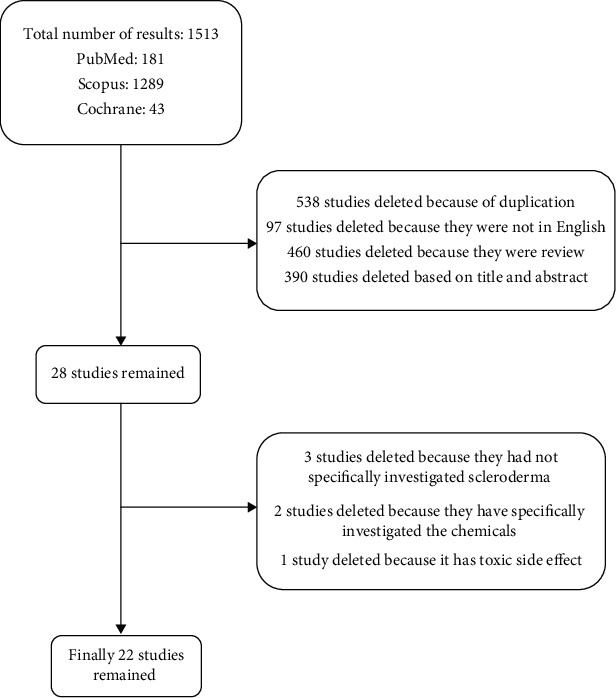
Study design diagram.

Data were collected by searching databases including PubMed, Scopus, and Cochrane Library, using search terms as “scleroderma” OR “systemic sclerosis” keywords in the title/abstract and “plant” OR “herb” OR “herbal preparation” OR “phytochemicals” keywords in the whole text. Search results were entered into the study regardless of time limitation; however, the final papers used in the study were from 1964 to 2020. Two researchers separately assessed the studies, and non-English, review, and duplicate articles were excluded from the study (Figure 2).

## 3. Results

From a total of 1513 studies, 390 studies were deleted based on their title and abstract, 538 studies were excluded due to duplication, and 460 reviews and 97 non-English studies were omitted. 28 studies remained for checking of the full text; therefore, 6 studies were deleted based on their full text. 34 other studies were further deleted due to irrelevancy to the criteria for the present study. Figure 2 represents the method of the study and criteria for article selection.

### 3.1. Medicinal Plants/Herbal Formulations for Scleroderma Treatment

**Figure 3:**
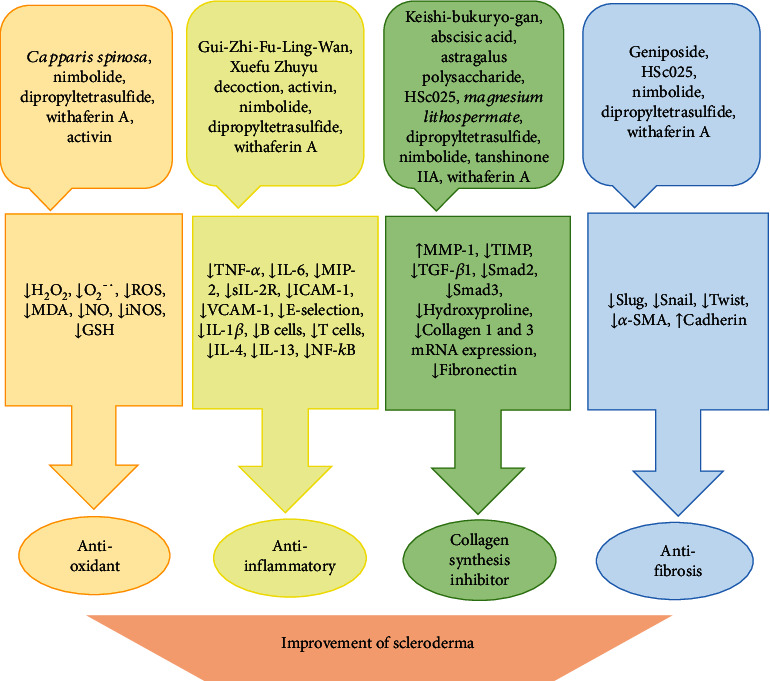
Mechanisms of action of herbal formulations and natural product for scleroderma treatment.

Here, we introduce some herbal formulations and their relevant mechanism of action that are beneficial for scleroderma treatment (Figure 3).

#### 3.1.1. *Capparis spinosa L.*

*Capparis spinosa* L. (Capparidaceae), common name caper, contains active compounds such as alkaloids, flavonoids, polyphenols, and sterols that are responsible for therapeutic properties of this plant-like anti-inflammatory, antioxidant, antiallergic, and antidiabetic [131]. *In vitro* investigation on systemic sclerosis and dermal fibroblasts showed that treatment with the ethanolic extract of *C.spinosa* reduced H_2_O_2_ and O_2_^−^ production, ROS level, and Ha-Ras expression and inhibited the phosphorylation of ERK/2. It was stated that the ROS-ERK1/2-Ha-Ras loop plays an important role in the pathogenicity of systemic sclerosis; therefore, *C.spinosa* can modulate systemic sclerosis by reducing oxidative stress and modulating the ROS-ERK1/2-Ha-Ras pathway [132] (Table 1).

#### 3.1.2. *Ginkgo biloba* L.

*Ginkgo biloba* L. (Ginkgoaceae), common name ginkgo, is a historical medicinal plant with a broad set of therapeutic actions [133, 134]. A high percentage of flavonoids and terpenoids is implicated in the pharmacological activities of *G. biloba* [135]. In a clinical trial on systemic sclerosis patients, consumption of *G. biloba* pills (120 mg/kg/day) for 3 months improved Raynaud's phenomenon such as reduction of attack duration and Raynaud's condition score. Raynaud's phenomenon is a complication of systemic sclerosis [136] (Table 1).

#### 3.1.3. Gui-Zhi-Fu-Ling-Wan (Keishi-Bukuryo-Gan)

Gui-Zhi-Fu-Ling-Wan (Keishi-bukuryo-gan) (GFW) is a herbal mixture consisting of 5 herbs including *Cinnamomum cassia* (L.) J. Presl (Lauraceae), *Wolfiporia extensa* (Pecks) Ginns (Polyporaceae), *Paeonia × suffruticosa* Andrews, *Paeonia lactiflora* Pall. or *Paeonia veitchii* Lynch (Paeoniaceae), and *Prunus persica* (L.) Batsch or *Prunus davidiana* (Carrière) Franch (Rosaceae). This mixture has many pharmacological applications in Chinese traditional medicine such as anti-inflammation, antioxidant, enhancement of the blood circulation, and improvement of scleroderma [137]. Coculture of fibroblast MRC-5 cells with GFW downregulated TNF-*α*, macrophage inflammatory protein 2 (MIP-2), and IL-6 mRNA expression and inhibited the proliferation of the fibroblasts [138]. Administration of GFW inhibited proliferation of human fibroblast cells and collagen synthesis *in vitro*, resulting in improvement of sclerosis of skin and internal organs. This treatment did not affect mRNA expression of collagen, so probably this inhibitory effect on collagen synthesis is in the posttranscriptional level [139] (Table 1).

#### 3.1.4. *Oenothera biennis* L.

*Oenothera biennis* L. (Onagraceae), commonly called evening primrose, is a plant used in herbal medicine because its oil has a high content of gamma-linolenic acid, a precursor to prostaglandin E1. Prostaglandin E1 increases blood flow and reduces inflammation, thus improving Raynaud's phenomenon and other defects associated with scleroderma [140]. In patients suffering from scleroderma, *O. biennis* oil treatment improved Raynaud's phenomenon, pain in hands and feet, ulcers, skin texture, and telangiectasia [141, 142] (Table 1).

#### 3.1.5. Persea americana Mill. (Avocado) and Glycine max (L.) Merr. (Soybean) Unsaponifiables

Avocado and soybean unsaponifiables are natural compounds made from one-third of avocado oil and two-thirds of soybean oil. Phytosterols, *β*-sitosterol, campesterol, stigmasterol, some vitamins, strolls, and terpenes are among the active components of this mixture. The therapeutic potential of this formulation was associated with the anti-inflammatory and antioxidant properties of its bioactive content. The mixture was found beneficial for osteoarthritis and another inflammatory disease [143]. It was reported that avocado and soybean unsaponifiables can restrain osteoarthritis through inhibition of proinflammatory cytokines such as IL-1*β*, IL-3, IL-6, IL-8, IL-13, and TGF-*β*; also, they can modulate oxidative damages by repression of ROS production [144–146].

In a study on 100 patients suffering from scleroderma, administration of 300 mg/kg/day of avocado and soybean unsaponifiables during 6 months reduced disability and deformity of these patients and improved the symptoms of the disease. It has been suggested that this herbal mixture can suppress the inflammatory mediators, inflammation, and cutaneous fibrosis, while enhancing collagen solubility and connective tissue regeneration, probably due to its rich antioxidant content [147]. Therefore, based on these studies, avocado and soybean unsaponifiables may be a candidate to alleviate scleroderma (Table 1).

#### 3.1.6. *Tripterygium wilfordii* Hook f.

*Tripterygium wilfordii* Hook f. (Celastraceae), Chinese name Lei Gong Teng, is a plant used in Chinese traditional medicine for many aims such as treatment of RA, systemic lupus, and systemic sclerosis. The plant contains more than 300 active compounds, of which diterpenoids, triptolide, tripdiolide, and triptomide were found effective in the treatment of autoimmune disease [148, 149]. Treatment with *T. wilfordii* improved forced vital capacity (FVC) and FVC percentages of predicted values (pred%) that are indicators of improvement of pulmonary function in systemic sclerosis patients [150] (Table 1).

#### 3.1.7. Xuefu Zhuyu Decoction

Xuefu Zhuyu decoction is a herbal decoction used in traditional Chinese medicine for enhancing blood circulation and treatment of atherosclerosis and neurodegenerative disease. Xuefu Zhuyu includes *Bupleurum chinense* DC. (Apiaceae), *Paeonia lactiflora* Pall. (Paeoniaceae), *Cyathula officinalis* K.C. Kuan (Amaranthaceae), *Conioselinumanthriscoides* “Chuanxiong” (Apiaceae), *Angelica sinensis* (Oliv) Diels. (Apiaceae), *Prunus persica* (L.) Batsch (Rosaceae), *Glycyrrhiza uralensis* Fisch. ex DC. (Fabaceae), *Carthamus tinctorius* L. (Asteraceae), *Platycodon grandiflorum* (Jacq.) A. DC. (Campanulaceae), *Rehmannia glutinosa* (Gaertn.) DC. (Orobanchaceae), and *Citrus × aurantium* L. (Rutaceae) [151]. Treatment of patients with localized scleroderma with vitamin B6 and Xuefu Zhuyu decoction reduced TNF-*α* and soluble interleukin 2 receptor (sIL-2R) levels in serum, possibly through the improvement of blood circulation and decrease of inflammation [152] (Table 1).

### 3.2. Natural Therapeutics for Scleroderma

Here, we introduce some natural therapeutics and their relevant mechanism of action that are beneficial for scleroderma treatment (Figure 3).

#### 3.2.1. Abscisic Acid

Abscisic acid is a phytohormone and also is an endogenous human hormone involved in many inflammatory processes of the body including the increase of the production of proinflammatory cytokines like TNF-*α*, MCP-1, matrix metalloproteinase 9 (MMP-9), and prostaglandin E2 (PGE2) and cell migration [153]. Abscisic acid treatment of Bruzzone et al. in human dermal fibroblasts enhanced proliferation of fibroblasts and decreased their migration, reduced the collagen level, and increased the MMP-1 and tissue inhibitor of metalloproteinase 1 (TIMP-1) levels. It is known that the expression of MMP-1 and TIMP-1 (MMP-1 inhibitor) reduces during the systemic sclerosis progression. Also, the plasma level of abscisic acid is lower in systemic sclerosis patients compared with normal people [154] (Table 2).

#### 3.2.2. Activin

Activin is a phytochemical derived from grape seed proanthocyanidins with strong antioxidant activity. The compound is used to improve cardiovascular disease associated with oxidative damage [155]. Treatment of activin (100 mg/kg/day) in systemic sclerosis patients for 30 days reduced soluble adhesion molecules (i.e., ICAM-1, VCAM-1, E-selectin) and malondialdehyde (MDA) levels in serum. E-selectin is a type of selectin molecule that plays an important role in the inflammation process and is activated by cytokines. Also, VCAM-1 and ICAM-1 are involved in inflammatory pathways. Therefore, this compound can modulate systemic sclerosis through attenuation of oxidative stress and reduction of adhesion molecules involved in inflammation [156] (Table 2).

#### 3.2.3. Astragalus Polysaccharides

Astragalus polysaccharide is a phytochemical derived from *Astragalus mongholicus* Bunge (Fabaceae) with great pharmacological activities, particularly in the treatment of cancer and modulation of inflammatory responses [157]. Administration of *Astragalus* polysaccharide in bleomycin-induced systemic sclerosis mice reduced the collagen production in skin tissue, also downregulating the TGF-*β*1, MCP-1, Smad2, and Smad3 mRNA expressions. As mentioned, activation of the TGF-*β*1/Smad2/3 pathway leads to an increase of the mRNA expression of collagen 1. Therefore, Astragalus polysaccharides can modulate systemic sclerosis by inhibiting collagen production [158] (Table 2).

#### 3.2.4. Bee Venom

Bee venom is used in traditional Chinese medicine for 3000 years with various health-promoting actions such as anti-inflammatory, antioxidant, antifibrotic, antiapoptotic, and antiatherosclerosis activities [159]. Bee venom modulated the number of itches and improved sleep index in scleroderma patients. The sleep index is defined as the numeric scale (NRS) score for itch and sleep [160] (Table 2).

#### 3.2.5. Curcumin

Curcumin treatment increased apoptosis in scleroderma lung fibroblast (SLF) but not in normal lung fibroblast (NLF), because protein kinase C (PKC) and heme oxygenase 1 (HO-1) and glutathione-*S*-transferase P1 (GST P1) are not active in SLF. Interestingly, the apoptotic effect of curcumin is specific to SLF, due to the activity of PKC in healthy cells and its inactivity in damaged cells. Overall, curcumin can modulate systemic sclerosis by increasing apoptosis in damaged cells [161] (Table 2).

#### 3.2.6. Dipropyltetrasulfide

Dipropyltetrasulfide is a natural compound derived from *Allium* spp. with high antioxidant, antiproliferative, and antibacterial activities and is used in folk medicine for the treatment of many diseases such as diabetes, cancer, and cardiovascular disease [162]. Administration of dipropyltetrasulfide in HOCl-induced systemic sclerosis mice reduced the expressions of *α*-SMA and pSmad2/3 in the skin tissue. Besides, dipropyltetrasulfide declined the count and proliferation of B and T cells in the spleen and the IL-4 and IL-13 levels in the serum of treated mice. *In vitro*, dipropyltetrasulfide reduced the proliferation of dermal fibroblasts and raised glutathione (GSH) levels. Modulation of the immune system, suppression of oxidative stress, and antifibrotic activity are the main mechanisms underlying the therapeutic property of dipropyltetrasulfide against systemic sclerosis [163] (Table 2).

#### 3.2.7. Geniposide

Geniposide is a phytochemical derived from *Gardenia jasminoides* J. Ellis (Rubiaceae) with a variety of pharmacological and biological activities such as antioxidant, anti-inflammatory, antidiabetic, antiproliferative, and neuroprotective actions [164]. In bleomycin-induced scleroderma mice, treatment of geniposide inhibited endothelial to mesenchymal transition (EndMT) process activity. In addition, geniposide increased the E-cadherin levels and Slug, Snail, and Twist protein expressions *in vivo*, while reducing *α*-SMA, phospho-mTOR, and phosphor S6 and enhancing cluster of differentiation 31 (CD31) in fibroblast cells *in vitro*. Slug, Snail, and Twist are transcriptional factors that regulate the expression of E-cadherin. Geniposide can attenuate systemic sclerosis via endothelial cell protection and inhibition of the mTOR pathway [165] (Table 2).

#### 3.2.8. HSc025

HSc025 is a novel small compound of trihydroxy-*α*-sanshool. Trihydroxy-*α*-sanshool is a compound derived from *Zanthoxylum piperitum* (L.) DC. (Rutaceae). Previous studies showed that the extract of this herb can inhibit collagen gene expression [166]. Hasegawa et al. demonstrated that treatment with HSc025 in human dermal fibroblasts reduced collagen expression. *In vivo*, HSc025 decreased the hypodermal thickness, hydroxyproline content, and the frequency of *α*-SMA-positive myofibroblasts in the skin of scleroderma mice. Moreover, HSc025 reduced lung fibrosis in these animals as measured by the Ashcroft score. HSc025 can be considered a treatment for systemic sclerosis by inhibiting TGF-*β*/Smad signaling and improving pulmonary fibrosis [166] (Table 2).

#### 3.2.9. Magnesium Lithospermate

Magnesium lithospermate is a compound derived from *Salvia miltiorrhiza* Bunge (Lamiaceae) with antioxidant, vasodilator, antifibrotic, improvement of blood circulation, and neuro- and cardiovascular-protective effects [167]. Shigematsu et al. demonstrated that treatment of human dermal fibroblasts from scleroderma patients with magnesium lithospermate reduced collagen synthesis by inhibition of prolyl and lysyl hydroxylase activities, indicating that magnesium lithospermate may be an antifibrotic drug for systemic sclerosis treatment [168] (Table 2).

#### 3.2.10. Nimbolide

Nimbolide is a phytochemical derived from *Azadirachta indica* A. Juss. (Meliaceae) and is used in Indian traditional medicine for the treatment of many diseases. It has a wide range of biological activities such as anticancer, antiarthritic, antifungal, antifibrotic, anti-inflammatory, antioxidant, and antigastric ulcer [169]. Treatment of nimbolide reduced skin thickness and oxidative stress in bleomycin-induced scleroderma mice. Nimbolide attenuated the progression of scleroderma by controlling inflammatory factors such as TNF-*α*, IL-1*β*, and p-NF-*κ*B and by downregulating the TGF-*β*/Smad signaling axis (inhibition of TGF-*β* expression and Smad2/3 protein phosphorylation). In addition, nimbolide inhibited the expressions of *α*-SMA and N-cadherin, indicators of epithelial to mesenchymal transition [170] (Table 2).

#### 3.2.11. Bromelain

Bromelain is a protein-digesting enzyme mixture derived from the stem, fruit, and juice of the pineapple, *Ananas comosus* (L.) Merr. (Bromeliaceae). It has been used for the treatment of inflammatory disease, pain, and muscle soreness in America [171]. In a case report study, it was shown that treatment of a systemic sclerosis patient with bromelain improved the patient condition by reducing depigmentation areas of the forehead and scalp, improving eating food, and improving hand and foot activities [172] (Table 2).

#### 3.2.12. Tanshinone IIA

Tanshinone IIA is a phytochemical derived from *S. miltiorrhiza*, which is used in traditional Chinese medicine for the treatment of oxidative damages, connective tissue diseases, cardiovascular diseases, systemic lupus erythematosus, systemic sclerosis, and inflammatory diseases like RA and pulmonary hypertension [173, 174]. Liu et al. demonstrated that treatment with tanshinone IIA in human dermal vascular smooth muscle cells (DVSMCs) reduced cell proliferation, collagen 1 and 3 expressions, migration, and ERK phosphorylation. The ERK/MAPK signaling pathway is an important pathway for systemic sclerosis progression, and tanshinone IIA can block this pathway [175] (Table 2).

#### 3.2.13. Withaferin A

Withaferin A is one of the active compounds derived from *Withania somnifera* (L.) Dunal (Solanaceae) and used in Indian traditional medicine with known applications for the treatment of immunological and inflammatory diseases [176]. Withaferin A reduced skin thickness and modulated the antioxidant parameters (reduced nitric oxide (NO) and inducible nitric oxide synthase (iNOS) and raised GSH levels) in supernatants of skin in bleomycin-induced systemic sclerosis mice. Withaferin A alleviated the E-cadherin, collagen, and hydroxyproline levels and the *α*-SMA and fibronectin expressions. Withaferin A treatment repressed the TGF-*β*1/Smad signaling pathway by inhibiting the expression of TGF-*β*1 and the phosphorylation of Smad2/3 protein. Besides, withaferin A downregulated the p-Akt, p-NF-*κ*B, p65, inhibitor of nuclear factor kappa-B (IKK*β*), TNF-*α*, and IL-1*β* expressions, while promoting the FOXO3a expression. Beneficial properties of withaferin A might be correlated with inhibition of inflammation, collagen synthesis, and oxidative stress in systemic sclerosis damaged tissue [177] (Table 2).

**Figure 4:**
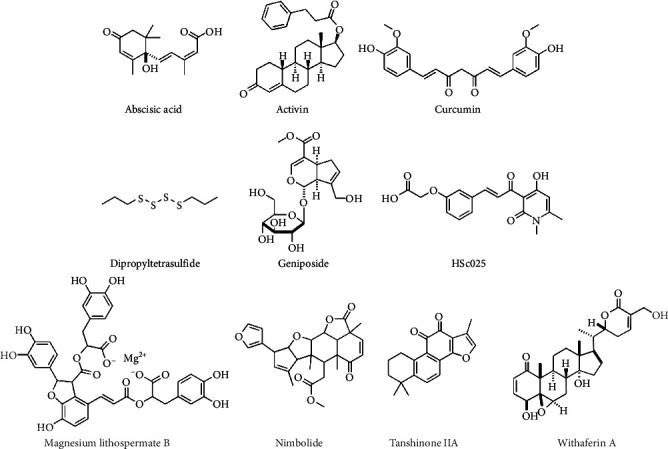
Chemical structures of selected natural product compounds with potential for scleroderma treatment.

Figure 4 shows the chemical structures of selected natural product compounds with potential for scleroderma treatment.

## 4. Discussion

Sclerodermas are rare autoimmune diseases, virtually affecting all the body tissues. Molecular analyses suggest that environmental and genetic factors may trigger the disease in vulnerable subjects. It was shown that both the innate and the adaptive immune systems are involved. Upon such condition, the number of autoreactive B cells producing autoantibodies and secretion of proinflammatory and profibrotic cytokines such as TGF-*β*, platelet-derived growth factor, connective tissue growth factor, IL-6, IL-4, and IL-1*α* by the immune, endothelial, and fibroblast cells increases, while T regulator cells are suppressed [178]. Yet, there is no definite cure; the lack of a uniform clinical-epidemiological approach, the necessity of long-term management in affected individuals, and adverse effects of current medications indicate an urgent need for new strategies for early diagnosis and to minimize the development of serious morbidity and to improve the quality of life for these patients. In this regard, natural products are a reliable source of new medicines, providing versatile lead structures to stimulate or prevent many pharmacological targets and activities. We provided evidence showing that natural preparations can improve systemic fibrosis mainly through suppressing inflammatory activities, inhibiting mitogen-induced lymphocyte proliferation, inducing cellular apoptosis targeting the mediators and signaling pathways of apoptosis, and eliciting T regulatory cells. However, these results are mainly based on research outcomes from Chinese pharmaceutical formulations, and to achieve a comprehensive conclusion, more evaluations and long-term clinical trials have to be conducted worldwide. Caution regarding the use of natural products/herbal formulations is still warranted since potential long-term consequences have not been evaluated.

## Figures and Tables

**Table 1:** *In vitro*, *in vivo*, and clinical interventions of medicinal plants for scleroderma treatment.

Herbal species and/or formulation	Type of study	Outcomes	Reference
*Capparis spinosa* L.	*In vitro*/systemic sclerosis dermal fibroblasts	↓H_2_O_2_, ↓O_2_^·^ˉ, ↓ROS, ↓cell death, ↓Ha-Ras, ↓ERK1/2	[132]
*Ginkgo biloba* L.	*In vivo*/clinical trial	↓Attack duration, ↓Raynaud's score	[136]
Gui-Zhi-Fu-Ling-Wan: *Cinnamomum cassia* (L.) J. Presl, *Wolfiporia extensa* Ginns, *Paeonia × suffruticosa* Andrews, *Paeonia lactiflora* Pall. or *Paeonia veitchii* Lynch, and *Prunus persica* (L.) Batsch or *Prunus davidiana* (Carrière) Franch.	*In vitro*/MRC-5 cells	↓TNF-*α*, ↓MIP-2, ↓IL-6	[138]
	*In vitro*/human fibroblasts	↓Proliferation, ↓collagen	[139]
*Oenothera biennis* L.	*In vivo*/clinical trial	↓Raynaud's score, ↓ulcers, ↓pain, ↓telangiectasia	[141, 142]
*Persea americana* Mill. (avocado) and *Glycine max* (L.) Merr. (soybean) unsaponifiables	*In vivo*/clinical trial	↓Deformity, ↓disability	[147]
*Tripterygium wilfordii* Hook f.	*In vivo*/clinical trial	↑FVC, ↑FVC pred%	[150]
Xuefu Zhuyu decoction *Bupleurum chinense* DC., *Paeonia lactiflora* Pall., *Cyathula officinalis* K.C. Kuan, *Conioselinumanthriscoides* “Chuanxiong,” *Angelica sinensis* (Oliv) Diels., *Prunus persica* (L.) Batsch, *Glycyrrhiza uralensis* Fisch. ex DC., *Carthamus tinctorius* L., *Platycodon grandiflorum* (Jacq.) A. DC., *Rehmannia glutinosa* (Gaertn.) DC., and *Citrus × aurantium* L. plus vitamin B6	*In vivo*/clinical trial	↓TNF-*α*, ↓sIL-2R	[152]

**Table 2:** *In vitro*, *in vivo*, and clinical interventions of natural therapeutics for scleroderma treatment.

Samples	Inducer	Type of study	Outcome	Ref.
Abscisic acid	—	*In vitro*/dermal fibroblast	↓Collagen, ↑MMP-1, ↑TIMP-1, ↓migration	[154]
Activin	—	*In vivo*/clinical trial	↓ICAM-1, ↓VCAM-1, ↓E-selectin, ↓MDA	[156]
Astragalus polysaccharide	BLM	*In vivo*/mice	↓Collagen, ↓TGF-*β*1, ↓MCP-1, ↓Smad2, ↓Smad3	[158]
Bee venom	—	*In vivo*/clinical trial	↓NRS for itch intensity, ↓NRS for sleep disturbance	[160]
Bromelain	—	*In vivo*/clinical trial	↓Depigmentation area, ↑food intake, ↑improvement of hand and foot activity	[172]
Curcumin	BLM	*In vivo/*mice *In vitro*/SLF	↑SLF apoptosis	[161]
Dipropyltetrasulfide	—	*In vivo*/mice *In vitro*/mouse dermal fibroblast	↓*α*-SMA, ↓AOPP, ↓pSmad2/3, ↓B cells, ↓T cells, ↓IL-4, ↓IL-13, ↑GSH	[163]
Geniposide	BLM	*In vivo*/mice *In vitro*/HUVECs	↓End MT, ↓*α*-SMA, ↓phospho-mTOR, ↓phospho-S6, ↓Slug, ↓Snail, ↓Twist, ↑CD31, ↑E-cadherin	[165]
HSc025	BLM	*In vivo*/mice *In vitro*/human dermal fibroblasts	↓Collagen, ↓hypodermal thickness, ↓hydroxyproline, ↓*α*-SMA, ↓fibrosis	[166]
Magnesium lithospermate	—	*In vitro*/human dermal fibroblast	↓Collagen	[168]
Nimbolide	BLM	*In vivo*/mice	↓Skin thickness, ↓NO, ↓TNF-*α*, ↓IL-1*β*, ↓p-NF-*κ*B, ↓TGF-*β*1, ↓Smad2/3, ↓*α*-SMA, ↓N-cadherin, ↑GSH	[170]
Tanshinone IIA	IL-17A	*In vitro*/DVSMCs	↓Proliferation, ↓collagen 1 and 3, ↓migration, ↓ERK phosphorylation	[175]
Withaferin A	BLM	*In vivo*/mice	↓Skin thickness, ↓E-cadherin, ↓*α*-SMA, ↓fibronectin, ↓hydroxyproline, ↓collagen, ↓p-Akt, ↓TGF-*β*1, ↓Smad2/3, ↓p-NF-*κ*B, ↓p65, ↓IKK*β*, ↓TNF-*α*, ↓IL-1*β*, ↓NO, ↓iNOS, ↑FOXO3a, ↑GSH	[177]

Abbreviations: AOPP = advanced oxidation protein products; BLM = bleomycin; HUVECs = human umbilical vein endothelial cells.
